# Mechanical, Thermal, and Fire Retardant Properties of Rice Husk Biochar Reinforced Recycled High-Density Polyethylene Composite Material

**DOI:** 10.3390/polym15081827

**Published:** 2023-04-09

**Authors:** Atta ur Rehman Shah, Anas Imdad, Atiya Sadiq, Rizwan Ahmed Malik, Hussein Alrobei, Irfan Anjum Badruddin

**Affiliations:** 1 Department of Mechanical Engineering, COMSATS University Islamabad, Wah Campus, Wah Cantt 47040, Pakistan; 2 Department of Mechanical Engineering, HITEC University, Taxila 47050, Pakistan; 3 Department of Metallurgy & Materials Engineering, Faculty of Mechanical and Aeronautical Engineering, University of Engineering and Technology, Taxila 47050, Pakistan; 4 Department of Mechanical Engineering, College of Engineering, Prince Sattam bin Abdullaziz University, AlKharj 11942, Saudi Arabia; 5 Department of Mechanical Engineering, College of Engineering, King Khalid University, Abha 61421, Saudi Arabia

**Keywords:** polymer composites, recycled high density polyethylene, rice husk biochar, mechanical properties, thermal properties

## Abstract

This study concentrated on the influence of rice husk biochar on the structural, thermal, flammable, and mechanical properties of recycled high-density polyethylene (HDPE). The percentage of rice husk biochar with recycled HDPE was varied between 10% and 40%, and the optimum percentages were found for the various properties. Mechanical characteristics were evaluated in terms of the tensile, flexural, and impact properties. Similarly, the flame retardancy of the composites was observed by means of horizontal and vertical burning tests (UL-94 tests), limited oxygen index, and cone calorimetry. The thermal properties were characterized using thermogravimetric analysis (TGA). For detailed characterization, Fourier transform infrared spectroscopy (FTIR) and scanning electron microscopy (SEM) tests were performed, to elaborate on the variation in properties. The composite with 30% rice husk biochar demonstrated the maximum increase in tensile and flexural strength, i.e., 24% and 19%, respectively, compared to the recycled HDPE, whereas the 40% composite showed a 22.5% decrease in impact strength. Thermogravimetric analysis revealed that the 40% rice husk biochar reinforced composite exhibited the best thermal stability, due to having the highest amount of biochar. In addition, the 40% composite also displayed the lowest burning rate in the horizontal burning test and the lowest V-1 rating in the vertical burning test. The 40% composite material also showed the highest limited oxygen index (LOI), whereas it had the lowest peak heat release rate (PHRR) value (52.40% reduced) and total heat release rate (THR) value (52.88% reduced) for cone calorimetry, when compared with the recycled HDPE. These tests proved that rice husk biochar is a significant additive for enhancing the mechanical, thermal, and fire-retardant properties of recycled HDPE.

## 1. Introduction

Global demand for fossil fuels is constantly increasing, but a huge challenge that the world has to resolve is their rapid depletion with the passage of time, along with the excessive pollution caused [1,2]. This is why people are shifting towards the use of renewable materials, particularly due to environmental concerns and the future scarcity of petroleum-based products [3]. On the other hand, the demand for food for the world’s population is increasing day by day, which has caused the accumulation of agricultural waste, and the most common way to get rid of this is to dispose of or incinerate it [4]. Disposing of this waste poses serious threats to environmental safety and hygiene. However, the biomass can be converted into products and energy [5]. Employing these various agricultural wastes in different composite materials could help alleviate this problem and represents a great solution for recycling and resource conservation [6]. Several types of organic waste, such as waste paper sludge and wool, have already been used to produce biocomposites [7,8,9,10]. However, researchers are now inclining towards the development of biochar from agricultural waste and utilizing it in composite materials, to improve the desirable mechanical, thermal, and fire-retardant properties. When any type of biomass is heated with an absence/limited supply of oxygen (pyrolysis), it will leave behind a porous carbonaceous material, with the volatile gases absent, thus resulting in biochar formation [11]. Biochar is composed of highly ordered turbostatically crystalline regions, accompanied by some random amorphous regions [12], owing to the presence of cellulose. This isotropic structure results in spaces that form a porous structure, just like a honeycomb structure [13]. The reviews carried out by Väisänen et al. and Mohanty et al. gave a fair idea of how effective biochar can be in a composite material [14,15].

Ayrilmis et al. analyzed the mechanical properties of a wood–plastic composite (Polypropylene with Maleic Anhydride-grafted Polypropylene and different mixtures of wood flour and charcoal flour) [16]. An investigation was performed to find the optimum biomass for biochar-based polypropylene composites with rice husk, course wool, coffee husk, and landfilled wood as biomasses and biochar made from landfill wood [17]. The wood-based composite exhibited the best mechanical properties, with rice husk coming second. Zhang et al. examined the incorporation of rice husk biochar into high-density polyethylene and then compared this with wood–plastic composites [18]. It was found that the biochar-reinforced plastic composites had much superior mechanical properties to the wood–plastic composites. Sundarakannan et al. reinforced sugarcane biochar with polyester resin [19]. The study also focused on the development of a biocomposite by reinforcing a cashew nut shell biochar with unsaturated polyester resin at different loadings [20]. Huber et al. investigated the effect of the particle size of miscanthus biochar on Polyamide polymer and found that particle size varied the mechanical properties of the composite to an appreciable extent [21]. Khan et al. studied the influence of biochar addition to epoxy resin and compared this with the addition of carbon nanotubes in an epoxy matrix [22]. The biochar-reinforced epoxy composites outperformed the carbon nanotube-reinforced epoxy composites, in terms of the mechanical properties. Bartoli et al. tested the mechanical characteristics of an epoxy-based composite with five different types of commercial biochar (rice husk, mixed softwood, miscanthus, oil seed rape, and wheat straw) [23]. Pandey et al. focused on the study of a biochar hybrid composite with sisal fiber, softwood biochar, and epoxy as resin [24]. Similarly, Ketabchi et al. carried out an analysis of varied amounts of biochar (made of oil palm empty fruit bunch fiber) in polypropylene/ethylene-vinyl acetate, to form a hybrid composite [25]. They found that 30% biochar was preferable for enhancing the mechanical properties without degrading the thermal properties. Zhang et al. evaluated the influence of rice husk biochar on the dynamic mechanical properties of a composite [26]. The study revealed that biochar had a positive effect on the creep resistance, dynamic viscoelasticity, and stress relaxation properties of the composite. However, as the testing temperature increased, the stress relaxation and creep resistance started to reduce. Bajwa et al. treated high-density polyethylene–wood fiber composites with maleic anhydride polyethylene (MAPE) and found an improvement in the mechanical properties of the biocomposite [27]. However, concern about the undesirable increase in the overall cost of production was also reported, owing to the expensive compatibilizers used.

Das et al. reported that the addition of biochar to a composite could increase the thermal stability and flame retardance to an excellent extent compared to the neat composite [1,2]. Time to ignition (TTI) and PHRR were significantly reduced with increased biochar contents, whereas the THR did not vary significantly. Das et al. also introduced wood and biochar into polypropylene, with two flame retardants (FRs): magnesium hydroxide (Mg(OH)_2_) and ammonium polyphosphate (APP) [28]. They concluded that less wood and a higher proportion of biochar was optimum for reducing flammability. Another research work by Das et al. considered the addition of wood-waste-derived activated biochar to polypropylene and revealed that the biochar was extremely effective in increasing the thermal stability and reducing the flammability of the PP. Dahal et al. converted wood pellets into biochar and then added this to epoxy. The study concluded that an increased biochar loading had a better tendency to retard fire [29]. Li et al. studied the addition of nano charcoal in polypropylene composites and found that an increase in the degradation temperature led to an increase in the overall thermal properties of the composites [30]. Zhang et al. also observed an enhancement in the thermal degradation temperature with rice husk biochar filled composites [31]. Bartoli and Arrigo et al. performed TGA on polylactic acid composites (PLA) [32]. They concluded that the addition of biochar increased the thermal properties of the composites, but at higher filler loadings, they started to decrease, due to agglomeration.

The quality of a biochar can adversely affect the mechanical properties of biocomposites. Das et al. stated that the most crucial factor in the performance of biochar-based composites, and what makes them so promising, is the carbon content and the surface area of the biochar [1,2]. Similar results were derived by Ho et al., in which they stated that a high surface area of biochar, which results from high temperature pyrolysis, usually assists in achieving the optimum dispersion of biochar particles in the polymer matrix [3]. Tomczyk et al. stated that increasing the pyrolysis temperature increased the carbon content and specific surface area of biochar [33]. Das et al. studied the quality of biochars made in different pyrolysis reactors [34]. They revealed that different types of pyrolysis reactors yielded different qualities of biochar, and the utilization of these reactors to produce biochar should be aligned with the intended application. However, among the operating conditions, temperature is most important, as it primarily controls the properties and quality, and adversely affects the yield of biochar produced during the process [35,36,37,38]. The effect of pyrolysis temperature on the quality of biochar was studied by Elnour et al., in terms of the morphological properties, physical properties, and biochar structure [39]. Zhang et al. also conducted research regarding the addition of rice husk biochar to high-density polyethylene, varying the pyrolysis temperature of the biochar [40]. Ayadi et al. investigated the effect of pyro-gasification temperature on the mechanical characteristics of biochar–polymer biocomposites [41].

The aim of this study was to achieve desirable properties of recycled HDPE close to those of virgin HDPE. For this purpose, particles of rice husk were converted into biochar. Three types of property were studied, i.e., mechanical, thermal, and fire retardant. Higher amounts of cellulose and proper dispersion of the rice husk biochar caused an enhancement in the mechanical properties of the recycled HDPE, whereas the formation of inorganic substances, and char layers increased the thermal and flame retardancy of the recycled HDPE. HDPE was found to fill the voids on the rough surfaces of biochar particles which has caused improvement in the mechanical properties, along with the improvement in the thermal and fire-retardant properties. Moreover, very little research has been done on recycled polymers. This works represents an attempt to move the recycled polymer industry into the limelight, so that more research will be carried out on this topic in the future.

## 2. Materials and Methods

Recycled HDPE pellets having a density of 0.96 g/cm^3^ and melt flow index of 0.55 g/10 min were obtained from NEWTECH pipes, Islamabad, Pakistan. The HDPE was recycled by the company from faulty pieces of pipe. It is a post-industrial material obtained from the crushing of HDPE pipes and re-extrusion. Rice husk with a bulk density of 0.12 g/cm^3^ was purchased from Allied Industry, Lahore, Pakistan.

The raw rice husk was crushed in a masala grinder (Geepas Masala grinder—RPM 4500) multiple times, to convert it into rice husk powder. The rice husk powder obtained from this masala grinder was then passed through a sieve of mesh size 50, to obtain a rice husk powder particle size of less than 300 µm. This rice husk powder was then put through the process of pyrolysis in a muffle furnace (Thermolyne Benchtop muffle furnace) at a temperature of 550 °C, in order to produce rice husk biochar. The pyrolysis temperature was increased from 25 °C (ambient temperature) to 550 °C, at a heating rate of 15 °C/min and maintained for 3 h under a 20 mL/min nitrogen environment.

The rice husk biochar and recycled HDPE were then dried under sunlight for 24 h. The recycled HDPE was mixed with rice husk biochar in a high-speed mixer for 30 min, to obtained the blends. To mix the composite blends, a micro twin-screw extruder (SHJ-Omega-20, Shanghai, China) with screw outer diameter of 19.8 mm and length/diameter ratio of 38:1 was used. Both zones of the extruder, i.e., the extruding zone and blending zone, were operated at 180 °C. 

The resulting pellets were then put in a manual injection molding machine, with the purpose of making the various samples for the mechanical and other types of test. The temperature of the manual injection molding was set at 185 °C. The percentage of rice husk biochar that was mixed with recycled HDPE was varied between 10% and 40%. These percentages with their nomenclature are shown in Table 1.

FTIR spectroscopy was carried out, in order to investigate and analyze the possible interactions between the matrix (Recycled HDPE) and filler (Rice husk biochar). This was performed with FTIR equipment (Scientific Nicolet 6700 model, Waltham, MA, USA), using a KBr disc method, with spectra ranging from 500 to 4000 cm^−1^ (32 scans at 4 cm^−1^ resolution).

Mechanical characterization of all the samples was performed using tensile, bending, and impact tests. The tensile test was performed using a Testometric Inc., Manchester, UK (Load cell: 100 kN), with reference to the D638-14 ASTM standard and a cross-head speed of 1.5 mm/s. A gauge length of 25 mm was used. The bending test was performed using a Testometric Inc., UK (Load cell: 100 kN), with reference to the D790-10 ASTM standard and a cross-head speed of 1.5 mm/s. A span length of 51 mm was used. The test was a three-point bending test. An Izod impact test was performed using a TM235, Bangkok, Thailand (Load arm: 16 kg Model) with reference to the D256-10 ASTM standard. The V-notch in the testing specimens was made using a milling machine (Hermle UWF, Gosheim, Germany). Mechanical tests were carried out on 5 samples for each composition. 

Scanning electron microscopy (SEM) was conducted with a JEOL JSM5910 (Tokyo, Japan) (maximum magnification: 300,000×; maximum resolving power: 2.3 nm) on specimens that had undergone tensile testing. The dispersion of the filler particles, possible interactions between the matrix and filler particles, as well as the reasons behind the variation in tensile properties were observed with a microscope.

The fire retardancy of all composite samples was checked using a horizontal and vertical burning tests, cone calorimetric tests, and limited oxygen index test (LOI test). Horizontal and vertical burning tests were carried out, in order to investigate the reaction-to-fire properties according to the UL-94 standards with reference to the D635-03 and D3801-19 ASTM standards. The dimensions of the samples for both tests were 125 × 13 × 3.6 mm. The composite burning time and rates were determined in a horizontal burning test, whereas the flame time, afterglow time, extent of after flame or afterglow up to the holding clamp, and dripping of cotton due to flaming drops for all types of composite specimen were analyzed in a vertical burning test. After the horizontal burning tests, the composites were awarded an HB rating based on the burning rate, and they were awarded V-0, V-1, or V-2 on the basis of the abovementioned parameters observed in the vertical burning test. Cone calorimeter (CC) tests were also performed, in order to evaluate the reaction-to-fire characteristics of the specimens using the E1354-17 ASTM standard. Samples of 100 × 100 × 3.2 mm were conditioned at 50% relative humidity and 23 °C. An external heat flux of 50 kW/m^2^ was used to determine the various flammable properties. The type of cone calorimeter employed for this purpose was from FTT Limited, East Grinstead, UK. The limited oxygen index (LOI) of all samples was determined using an oxygen index tester. The standard ASTM D 2863-17 was followed, with a sample size of 100 mm × 6.5 mm × 3.2 mm. This was performed in a Sataton Limited oxygen index tester. The flow rate of oxygen and oxygen–nitrogen mixture were 3 L/min and 20 L/min, respectively.

Thermal stability was investigated in a Perkin Elmer, Waltham, MA, USA (Pyris Diamond Series TG/DTA model) using TG and DTG curves. This was carried out by heating the sample at a constant rate in an inert atmosphere. Heating was performed at a rate of 20 °C/min at a flow rate of 35 mL/min in an inert atmosphere of nitrogen. Weight loss vs. temperature plots were recorded as a result of the TGA. Similarly, in the case of DTG, the rate of material loss (weight loss) with heating was plot against the temperature and used to simplify the readings of the TG curves, which were quite close together.

## 3. Results

**Figure 1:**
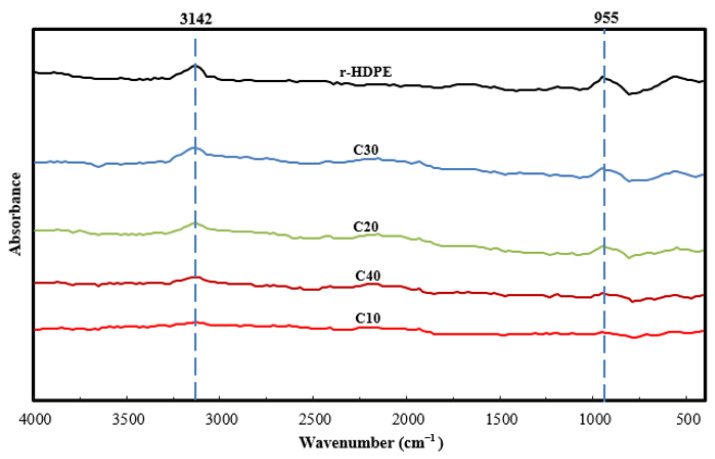
FTIR of recycled HDPE and all composites.

The FTIR spectra of the composite samples, as well as the recycled HDPE and rice husk biochar, are shown in Figure 1. Different peaks were observed at different wavenumbers, which are shown in Table 2. However, the intensity of these peaks varied from sample to sample. C-H stretching vibration, which is attributed to aliphatic structures, was responsible for the peak at 2924 cm^−1^ [42]. Similarly, the peak at 1492 cm^−1^ reflected C-H bending, which is a sign of alkanes [31]. However, if we focus on the wavenumber 955 cm^−1^, peaks can be observed for the composite samples, but no peak is observed for the recycled HDPE sample. These peaks in the biochar-derived composite sample were actually caused by C-O stretching vibration in the composite samples only, which indirectly indicates a lack of C-O stretching vibration in the recycled HDPE [43]. This reveals that the peak at this wavenumber was caused by a functional group that is only present in rice husk biochar, similarly to the presence of carbohydrates only found in rice husk. Similarly, the minor peak at 3611 cm^−1^ is related to H_2_O, which is caused by O-H bond stretching vibration, and was weakly detected and only contributed by the biochar [44,45,46,47]. The peak at 1614 cm^−1^ demonstrates C=C stretching [48], whereas that at 778 cm^−1^ represents the silica functional group [49]. Both of these groups were also contributed by the rice husk biochar. The above study also showed that these functional groups were either contributed by the recycled HDPE or rice husk biochar, without any new functional groups being present. This revealed the potential of the physical combination of HDPE and rice husk biochar.

**Figure 2:**
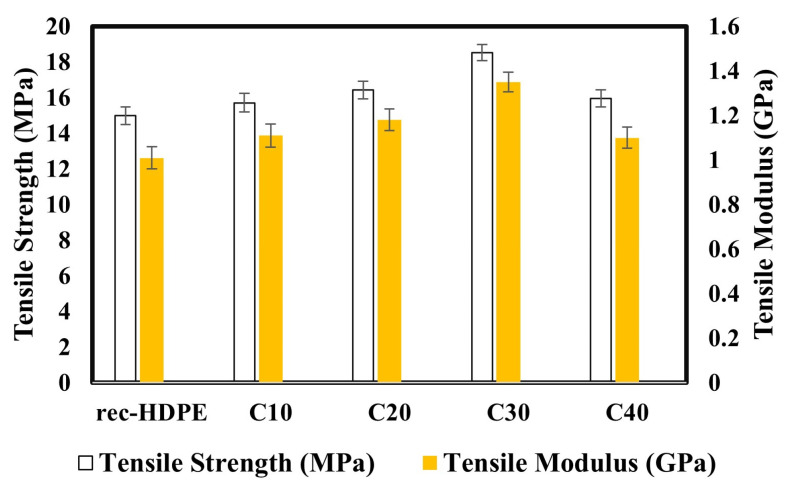
Tensile properties of the recycled HDPE and composites.

Figure 2 compares the tensile properties of the rice husk biochar derived composite samples with the recycled HDPE. The tensile strength of recycled HDPE was found to be 14.99 MPa. The tensile strengths of C10, C20, C30, and C40 were 15.71, 16.43, 18.54, and 15.97 MPa, respectively. The maximum tensile strength was exhibited by C30. At C30, a 24% increase in tensile strength was observed, compared to the 14.99 MPa of recycled HDPE. Regarding the elastic modulus, the elastic modulus of the recycled HDPE was found to be 1.01 GPa, and the elastic moduli of C10, C20, C30, and C40 were 1.11, 1.18, 1.35, and 1.1 GPa, respectively. The maximum elastic modulus was displayed by C30, which showed an 34% enhancement in tensile modulus.

The mechanical properties of composites are dependent on the homogeneous dispersion of filler particles in the matrix and the extent of agglomeration of the particles with each other. Another factor that reflects the tensile strength of a composite is the stress transfer mechanism of interfacial bonding [50]. If the recycled HDPE uniformly and effectively filled the pores of the biochar and transferred the stress productively, then the chance of an increase in mechanical properties was extremely high, as this would provide rigidity to the composite.

With reference to our case, the high tensile strength of the 30% rice husk biochar derived composite could be attributed to the uniform and homogenous dispersion of biochar in the HDPE matrix, less agglomeration of particles, and a good stress transfer mechanism, which was caused by good interfacial bonding, due to proper filling of the biochar pores by the recycled HDPE, which is also shown in the SEM image of C30. With the addition of 40% biochar, the tensile strength decreased because of the non-uniform dispersion of biochar particles, greater agglomeration of particles, and weak interfacial bonding [51]. Regarding the elastic modulus, biochar particles are subject to less deformation and are extremely rigid. On account of this property, the mobility of the recycled HDPE macromolecules was severely restricted, and this led to an increase in elastic modulus with the addition of biochar particles to the recycled HDPE, from 0 to 30% [52]. The elastic modulus of the C40 might have been low due to the agglomeration of particles and poor stress transfer between the filler particles and matrix. The SEM images further illustrate the causes of the variation in the tensile properties.

**Figure 3:**
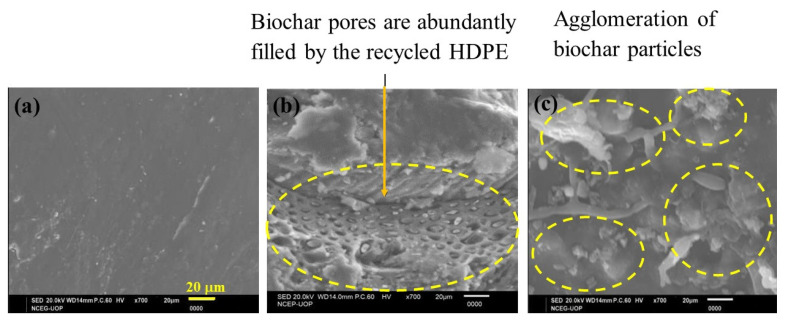
SEM images of (**a**) unmodified recycled HDPE, (**b**) 30% rice husk reinforced recycled HDPE, and (**c**) 40% rice husk reinforced recycled HDPE.

SEM images of the tensile cross-sectional fracture surface of the recycled HDPE, as well as the rice husk biochar composites, are shown in Figure 3. The smooth structure of recycled HDPE is shown in Figure 3a. For the 30% rice husk reinforced composite, i.e., in the case of C30, the porous structure of biochar was evident, as can be seen in Figure 3b. Most of the pores of the biochar were uniformly filled by the recycled HDPE. The better distribution of the filler particles can be viewed in this figure. This led to a good physical–mechanical interlocking structure [44,45,46]. This superior dispersion also caused an efficient transfer of stress between the rice husk biochar particles and recycled HDPE. This phenomenon revealed that the highest tensile strength among all samples was for C30. In the SEM image of C40 in Figure 3c, there are some signs of agglomeration and clusters of rice husk biochar, which lowered the tensile strength.

Furthermore, C30 also displayed the highest tensile modulus, due to the having the most interlocking of biochar pores with the recycled HDPE, which gave it rigidity and stiffness. C10 displayed the lowest tensile modulus, owing to the lower rice husk biochar loading, whereas the agglomeration in C40 also caused a decreased tensile modulus.

**Figure 4:**
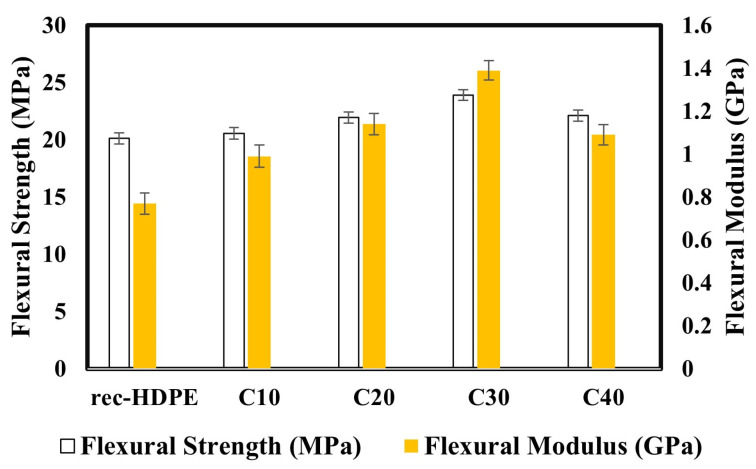
Flexural properties of the recycled HDPE and composites.

Figure 4 compares the flexural properties of the recycled HDPE with the biochar-derived composite samples. The flexural property of the recycled HDPE was discovered to be 20.12 MPa. The flexural properties of C10, C20, C30, and C40 were 20.56, 21.94, 23.91, and 22.12 MPa, respectively. C30 demonstrated an enhancement of 19% in flexural strength compared with the recycled HDPE and showed the maximum flexural strength. Moreover, C30 also recorded an increase of 80% for the flexural modulus compared with the recycled HDPE, which displayed a 0.77 GPa flexural modulus. C10, C20, C30, and C40 displayed 0.99, 1.14, 1.39, and 1.09 GPa flexural moduli. The trend in the flexural properties was found to be similar to that of the tensile properties.

This enhancement in flexural properties was due to the porous structure of the biochar and the physical interlocking between the biochar and recycled HDPE. When screw extrusion was performed, the HDPE (which was in a fluid state) could fill the pores of the biochar, so that a rigid structure with a good interaction between them was achieved; and after cooling, a strong physically interlocking structure was secured [11].This was the main reason behind the enhancement in the flexural properties with the addition of biochar to the recycled HDPE, since it allowed transferring the stress efficiently. Another factor responsible for the variation in the flexural properties, and reported in many documents, was the good particle dispersion in the matrix [53,54]. This could also have been the reason behind the decrease in the flexural strength and flexural modulus in the C40 composite. The biochar particles may not have been uniformly dispersed in the HDPE matrix or these particles might have become agglomerated, as a result of which there was appreciable reduction in the flexural characteristics.

**Figure 5:**
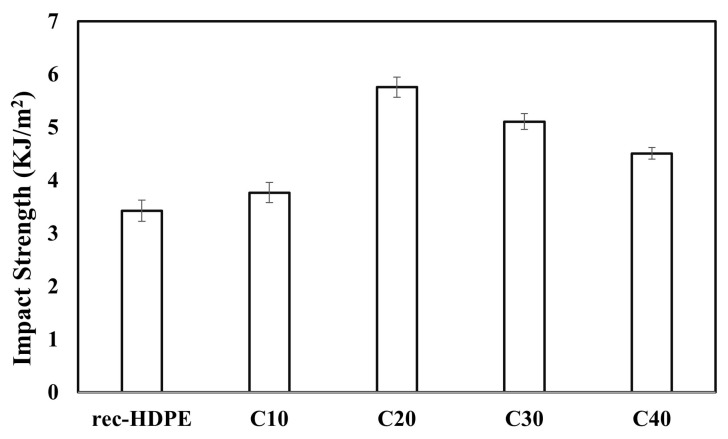
Impact properties of the recycled HDPE and composites.

The impact strengths of the recycled HDPE and composite materials are shown in Figure 5. The impact strength of the recycled HDPE, C10, C20, C30, and C40 were 3.43, 3.77, 5.76, 5.11, and 4.51 KJ/m^2^, respectively. The impact strengths all displayed a different trend compared to the tensile strength and flexural strength. Its decrease was consistent with the fiber–polymer composites [18,55]. The decrease of the impact strength from the rec-HDPE to the C40 sample was around 22.5%.

The impact strength of a composite material is entirely dependent on the rigidity and toughness [31]. The lesser the rigidity of the material, the greater its toughness, and the greater its impact strength, and vice versa. The rigidity of the materials was dependent on the mobility of the recycled HDPE molecules, which in turn was dependent on the physical–mechanical interlocking structure of the biochar with the HDPE molecules. When the content of the rice husk biochar in the recycled HDPE matrix was 10%, there was less chance of the recycled HDPE flowing into the pores of biochar, and thus the mobility of the recycled HDPE was very high. Its ability to absorb energy during fracture propagation was excellent, and thus its toughness was high. There were less regions of stress concentration, which required less energy to induce cracks in the composite, and thus the impact strength was very high. However, as the content of rice husk biochar was increased from 20% to 40%, the recycled HDPE started filling the pores of the biochar and the ability of the recycled HDPE to move began to be limited. This caused the increase in the rigidity of the composite and decrease in the impact strength of the samples [31]. As such, less energy was required to resist a sudden impact as the content was increased to 40%. Hence, the impact energy started to decrease. Another reason might have been that there were abundant regions of poor stress concentrations, and this led to easier crack propagation in the composite samples [31]. Table 3 compares the mechanical properties of the samples.

Horizontal and vertical burning tests were conducted on all specimens, i.e., with and without the inclusion of the rice husk biochar in the recycled HDPE. The results were then compared with the UL-94 standard for horizontal and vertical burning tests. For both the tests, five specimens for each sample were tested, and the average of the results was taken. In the case of the horizontal burning test, the time needed to reach from 25 mm to 100 mm (burning time) was increased as the content of rice husk biochar in the recycled HDPE was increased. This caused a decrease in the burning rate from the recycled HDPE to C40. All the specimens received an HB rating based on the burning rate, except the recycled HDPE sample, whose burning rate was greater than 40 mm/min and, thus, not awarded an HB rating. The decrease in the average burning rate from the recycled HDPE (45.10 mm/min) to C40 (25.99 mm/min) was 42.37%.

In the case of the vertical burning test, the flame and afterglow times after the 1st and 2nd flame applications progressively decreased with the increase in the biochar loading, from C20 to C40. This was why the recycled HDPE did not receive a rating, due to its poor flame retardancy characteristics. C10 also did not obtain a rating, due to the small amount of biochar. The after-flame time for C10 was greater than for the recycled HDPE, because the content of biochar was less. The rice husk biochar partially slowed down the flame speed but could not stop it, and the flame eventually reached the holding clamp. C20 obtained the rating of V-2, owing to the relatively high content of biochar, which resisted the flame and did not allow it to reach the holding clamp. C30 and C40 obtained a V-1 rating, owing to the shorter after flame and afterglow times, during which the rice husk biochar acted against the flame, slowed down the process of combustion, and stopped the flame before it reached the holding clamp. Cotton dripping was also not recorded with the C20, C30, and C40 samples. Finally, C40 had the lowest chance of burning. It is expected that, following this trend, recycled HDPE having 50% biochar would achieve a rating of V-0. Table 4 compares the fire-retardant properties of all samples.

**Figure 6:**
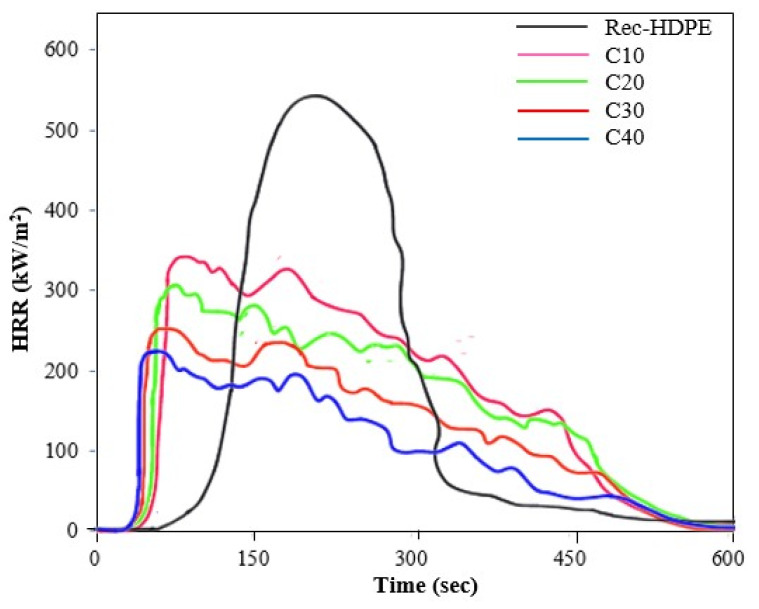
Heat release rate of the recycled HDPE and composites.

**Figure 7:**
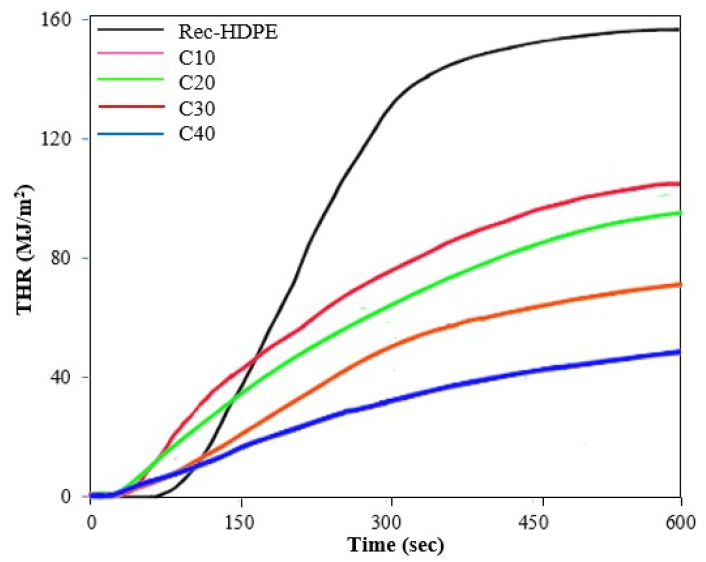
Total heat release rate of the recycled HDPE and composites.

Cone calorimeter tests were performed for the purpose of elaborating the principal fire retardant characteristics of the recycled HDPE, as well as the rice husk biochar derived composite samples. Figure 6 and Figure 7 show the HRR and THR curves of all samples with the response time, whereas Table 4 displays the time to ignition (TTI), time to peak heat release rate (TPHRR), heat release rate (HRR), and total heat release rate (THR) of all samples.

Time to ignition (TTI) is one of the crucial factors that dictates the flammable characteristics of composites. The recycled HDPE took some time to ignite and catch fire (59 s), whereas the TTI of the rice husk biochar derived samples first decreased for C10 and C20 and then increased for C30 and C40 as the content of the biochar increased. This means that the rice husk biochar samples were ignited in less time compared to the recycled HDPE. This decrease in TTI was immediate when the biochar was added to the composites, i.e., from the recycled HDPE to C10. This decrease in TTI with the addition of biochar was consistent with the report of Das et al. [11]. Similarly, the peak heat release rate (PHRR) and total heat release rate (THR) are also critical factors in judging the fire-retardant characteristics of composites and describe the overall combustion behavior of the material [56]. On the other hand, TPHRR tells us about the time it takes to produce the maximum amount of heat. The peak heat release rate (PHRR) and total heat release rate (THR) of the recycled HDPE were highest at 633 kW/m^2^ and 144 MJ/m^2^, due to its poor flammable properties and the intense combustion, whereas both of these properties showed a decline with the addition of rice husk biochar to the composite samples, with the 40% rice husk biochar derived composite sample displaying the lowest PHRR (301 kW/m^2^) and lowest THR (68 MJ/m^2^). 

This meant that C40 exhibited a large decrease of 52.40% in PHRR value and 52.88% in THR value. C40 also had the longest TPHRR (233 s) among all samples, which is also a positive attribute. All these desirable shifts in properties occurred with the increase in the content of biochar in the composite samples. This means that C40 displayed the best flammable properties, and the trend in best fire-retardant properties was as follows:

C40 > C30 > C20 > C10 > Recycled HDPE

The presence of biochar, which also possessed excellent thermal properties, acted as a hindering agent and became a barrier to the transfer of heat between the recycled HDPE and the source of heat [11,17]. Moreover, the formation of char layers from the rice husk biochar played a pivotal role in reducing the HRR and PHRR values [11].

Limited oxygen index (LOI) tests were performed, for the purpose of elaborating and further characterizing the flame retardancy behavior of the recycled HDPE as well as the biochar-derived composite samples. The greater the LOI of a specimen, the better its flame retardant properties. The limited oxygen index basically refers to the volume ratio of oxygen in a mixture of nitrogen and oxygen. It is the minimum concentration of oxygen that is required to sustain a flame or to support the combustion of a material.

**Figure 8:**
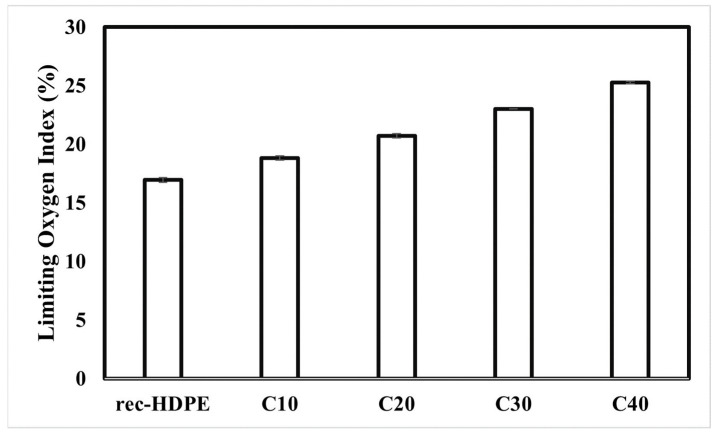
Limited oxygen index of the recycled HDPE and composites.

With reference to the LOI of the recycled HDPE and the biochar-derived samples, as shown in Figure 8, the LOI of the recycled HDPE was the lowest, whereas it increased as the content of rice husk biochar in the composite samples was increased.

The recycled HDPE showed the LOI of 16.97%, whereas the LOI of C40 was 25.28%, which was highest among all samples. This trend of the enhancement in LOI is consistent with previous reports [44,45,46]. This means that an increase of 48.97% was recorded in the LOI from the recycled HDPE to C40. The prime reason behind this was the presence of biochar, which enhanced the flame-retardant properties of the composites. Biochar has a very high thermal stability, so inclusion of biochar repelled the heat transport between the source of heat and the recycled HDPE [11]. It was also reported in the literature that, during the process of pyrolysis or carbonization, many metal oxides and inorganic substances are formed, which have the ability to slow down the process of combustion, as they are nonflammable [57,58,59].

**Figure 9:**
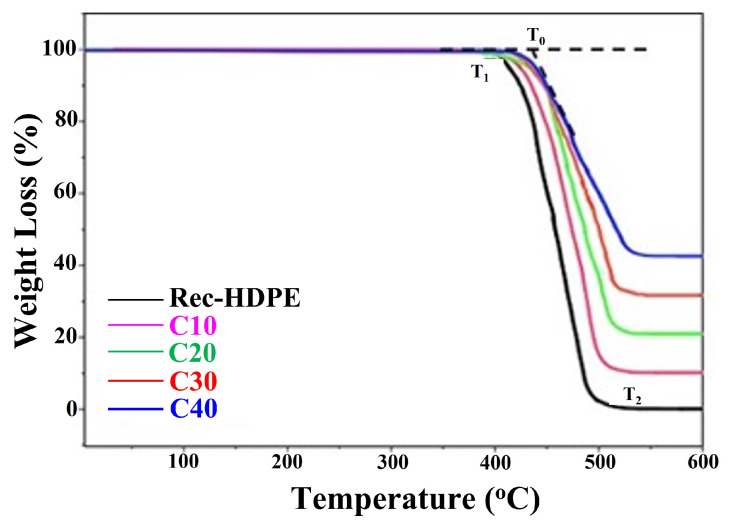
TG curves of the recycled HDPE and composites.

**Figure 10:**
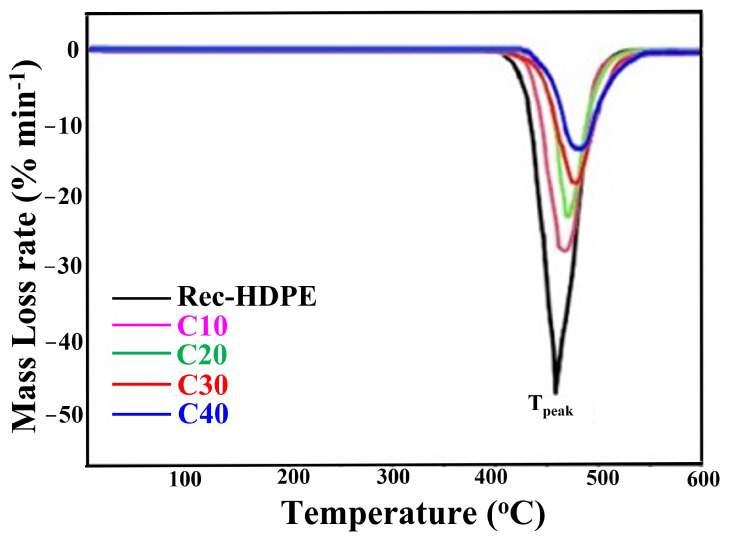
DTG curves of the recycled HDPE and composites.

Thermogravimetric and derivative thermogravimetric curves are shown in Figure 9 and Figure 10. The TG curves depict the percentage weight loss of the samples, whereas the DTG displays the derivative of those curves. These curves show that, as the heating temperature was increased, the samples initially responded well, with no degradation, but as the temperature reached 402.34 °C, the recycled HDPE sample started to thermally degrade, with degradation occurring at 522.16 °C. As the heating temperature was further increased, all samples started to degrade one by one, with C40 degraded last. We can also notice from the TGA that, as the biochar content in the composite samples was increased, the delay in thermal degradation increased, and this delay increased as the biochar content in the recycled HDPE was increased. This means that the increase in the biochar loading was extremely important in repelling the heat from decomposing the composite samples and in increasing the time of decomposition. In addition, the onset degradation temperature (T_0_) also rose with the increase in rice husk biochar loading. This increase was recorded as 4.05% from the recycled HDPE (425.09 °C) to C40 (442.36 °C). Table 5 shows the onset degradation temperature (T_0_), start degradation temperature (T_1_), finish degradation temperature (T_2_), point of inflection temperature (T_peak_), and residue % at 600 °C.

Moreover, if we look at the TG curves, we come to the conclusion that the amount of residue left after heating was directly proportional to the biochar loading. Das reported that this increase in residue generation is due to the increase in stable biochar loading [11]. De Bhowmick et al. also reported that the main reason behind the increase in the residue is the enrichment of stable SiO_2_ in stable biochar loading [57]. This is why the recycled HDPE was almost completely decomposed after heating and why this decomposition decreased as we moved towards higher loadings of biochar. C40 retained the maximum residue after heating, owing to a greater percentage of biochar. This increase in residues at 600 °C also proved the fact that the thermal stability was improved with the addition of biochar. The following trend of residue retention was followed by the samples:

C40 > C30 > C20 > C10 > Recycled HDPE

Hence, we come to the conclusion that biochar is an extremely important additive for increasing the thermal stability of composites.

## 4. Conclusions

The influence of variation of the rice husk biochar content in recycled HDPE was evaluated, in order to maximize the use of agricultural waste. All functional groups were supplied either by the recycled HDPE or rice husk biochar, which was revealed using FTIR.

The 30% biochar loading had the highest tensile strength (18.54 MPa), tensile modulus (1.35 GPa), flexural strength (23.91 MPa), and flexural modulus (1.39 GPa). The tensile and flexural strength were increased by 24% and 19%, respectively, compared with the recycled HDPE, whereas the tensile and flexural moduli were improved by 34% and 80%, respectively. However, the impact strength decreased (22.5%) when adding rice husk biochar to the recycled HDPE, due to the increase in rigidity of the composites compared with the recycled HDPE (4.63 KJ/m^2^). This variation in properties was due to the extent of agglomeration, level of homogeneous dispersion of the filler particles in the matrix, and the extent of the recycled HDPE filling the pores of the rice husk biochar, which was also displayed in the SEM images. Overall, this revealed that the addition of rice husk biochar had a positive effect on the mechanical properties of the composites.

With the increase in the content of rice husk biochar, the delay in thermal decomposition was enhanced, the ability to repel the heat from decomposing the composite samples was increased, and the time of decomposition was also increased. The C40 composite with 40% rice husk biochar displayed the best thermal stability and longest thermal decay time among all samples. Moreover, C40 showed an increase of 4.05% in the temperature of the onset of degradation compared to the recycled HDPE.

The composite with 40% rice husk biochar demonstrated the best flame retardancy characteristics. The horizontal burning test revealed a decrease in the burning rate of the recycled HDPE from 45.10 mm/min to 25.99 mm/min for C40. The vertical burning test also showed a change in the flammability rating, from the no rating of recycled HDPE to the V-1 of C40. The limited oxygen tests also showed that C40 had the highest LOI (25.28%). Moreover, the cone calorimetry also showed a decrease of 52.40% for the PHRR value and 52.88% for the THR value compared with the recycled HDPE. This was due to the inherent capacity of biochar to halt the transport of heat from the heat source to the matrix, as well as due to the formation of inflammable metal oxides and inorganic substances during pyrolysis.

## Figures and Tables

**Table 1:** Nomenclature of the samples.

Sr. No.#	Recycled Pellets wt. %	Rice Husk Biochar wt. %	Nomenclature
1	100	0	Rec-HDPE
2	90	10	C10
3	80	20	C20
4	70	30	C30
5	60	40	C40

**Table 2:** Functional groups in the composites and filler particles, corresponding to their wavelength.

Material	Wavelength (cm^−1^)	Bonds
Recycled HDPE	2924	C-H Stretching of Hydrocarbons
	1492	C-H Bending of Alkanes
Rice husk biochar	3611	O-H Stretching of Phenolic hydroxyl and Alcohol hydroxyl
	1614	C=C Stretching of Hydroxyl Functional groups
	955	C-O Stretching of Carbohydrates
	778	Silicon Hydrogen Single Bond (Si-H)
C10,C20,C30,C40	3611	O-H Stretching of Phenolic hydroxyl and Alcohol hydroxyl
	2924	C-H Stretching of Hydrocarbons
	1614	C=C Stretching of Hydroxyl Functional groups
	1492	C-H Bending of Alkanes
	955	C-O Stretching of Carbohydrates
	778	Silicon Hydrogen Single Bond (Si-H)

**Table 3:** Mechanical properties of the recycled HDPE and composites.

Samples	Tensile Strength (MPa)	Tensile Modulus (GPa)	Flexural Strength (MPa)	Flexural Modulus (GPa)	Impact Strength (kJ/m^2^)
Rec-HDPE	14.99	1.01	20.12	0.77	3.43
C10	15.71	1.11	20.56	0.99	3.77
C20	16.43	1.18	21.94	1.14	5.76
C30	18.54	1.35	23.91	1.39	5.11
C40	15.97	1.10	22.12	1.09	4.51

**Table 4:** Fire retardant properties of the recycled HDPE and composites.

Material Types	Cone Calorimetric Test				Horizontal Burning Test					Vertical Burning Test				LOI Test
	TTI (s)	TPHRR (s)	PHRR (kW/m^2^)	THR (MJ/m^2^)	Avg. Burning Time (min)	Avg. Burning Rate (mm/min)	Rating	Max. After Flame Time (s)	Total After Flame Time (s)	Max After Flame + After Glow Time (s)	Flame up to the Holding Clamp	Cotton Ignited by Flaming Drops	Rating	LOI (%)
Rec-HDPE	59	210	633	144	1.66	45.10	Nil	61	281	-	Yes	Yes	Nil	16.97
C10	32	214	434	120	2.01	37.19	HB	63	304	-	Yes	Yes	Nil	18.82
C20	28	222	399	103	2.30	32.66	HB	26	235	34	No	Yes	V-2	20.73
C30	30	229	344	83	2.64	28.38	HB	16	152	22	No	No	V-1	23.01
C40	31	233	301	68	2.89	25.99	HB	7	59	8	No	No	V-1	25.28

**Table 5:** Thermal properties of the recycled HDPE and composites.

Type of Sample	T_0_ (Onset Degradation Temperature) (°C)	T_1_ (Start Degradation Temperature) (°C)	T_2_ (Finish Degradation Temperature) (°C)	T_peak_ (Point of Inflection Temperature) (°C)	Residue % at 600 °C
Rec-HDPE	425.09	402.34	522.12	459.63	0.39%
C10	435.86	406.12	528.05	466.41	10.95%
C20	440.93	410.87	535.67	471.18	21.47%
C30	438.14	416.03	541.31	477.35	32.09%
C40	442.36	421.21	548.88	481.22	43.51%

## Data Availability

All data are included in the manuscript.
